# To What Factors Do Rural-Dwelling Hispanics Attribute Depressive Symptoms?

**DOI:** 10.1155/2013/781986

**Published:** 2013-05-26

**Authors:** Ipsit V. Vahia, Alvaro Camacho, Colin A. Depp, Angelica Herrera, Wesley K. Thompson, Rodrigo Munoz, Dilip V. Jeste, Bernardo Ng

**Affiliations:** ^1^Department of Psychiatry, University of California, San Diego, CA 92093, USA; ^2^Sun Valley Behavioral & Research Center, Imperial, CA 92251, USA; ^3^Sam and Rose Stein Institute for Research on Aging, University of California, San Diego, La Jolla, CA 92093, USA; ^4^University of Maryland, Baltimore County (UMBC), Baltimore, MD 21250, USA

## Abstract

This study is a retrospective chart review comparing rural-dwelling Caucasian and Hispanic outpatients' attribution of depressive symptoms. Based on the data gathered at intake, Hispanics were more likely to attribute depression to curse/spell and supernatural causes, while Caucasians were more likely to attribute symptoms to hereditary factors or job stress. Among both groups, higher CESD score was associated with problems with significant others or how they got along with others. Among Hispanics, depression severity was additionally associated with problems related to job or finances. Our findings point to a consequential role for clinical inquiry into attributed causes of depressive symptoms.

## 1. Introduction

Identification and adequate treatment of mood spectrum disorders, particularly depression and subsyndromal depression, is a major public health concern [1]. Recent data from the 2010 US Census showed that Hispanics are the largest and fastest growing minority group in the USA, and will have considerable impact on the delivery of services, treatment, and healthcare costs [2]. Moreover, it has been suggested that the prevalence of depression in Hispanics (especially immigrants and less acculturated persons) may be higher than the general population [3, 4]. Little, however, is known about how cultural differences and attributions of cultural beliefs impact depression [3], especially among Mexican-Americans. Even less is known about rural populations of Hispanics, though they represent a large group [5].

It is unclear if existing explanatory models of attribution (e.g., the health belief model or the explanatory model of illness) apply to Hispanics [6], and studies on the role of culture in depression among Mexican-Americans [7] have focussed more on adapting to American culture, rather than the role of culture-related beliefs such as superstitions or beliefs in the supernatural. In addition, there is little in the existing literature on the interaction of beliefs about mental illness with socioeconomic factors or the possible impact of beliefs about illness on care-seeking. However, theoretical models indicate that cultural meanings and norms about mental illness are likely to influence expression, and subsequently diagnosis and treatment for psychiatric conditions [8]. In light of the increase in the population, the number of Hispanic patients who have mental health problems will increase, and thus there is an urgent need to broaden our understanding of factors that influence what Hispanics attribute as the cause of depressive symptoms, and how culture may have a role. 

Imperial County is a large rural and semirural region of Southern California that provides an opportunity to study the attribution of depressive symptoms, socioeconomic stressors, acculturation, and depression among Mexican-Americans. Imperial is the poorest county in California, with 18% of their residents living below poverty line and 80% of the population being of Hispanic origin, in large part due to the proximity to the U.S.-Mexico Border [9]. Furthermore, as of the year 2010, this county has the highest unemployment rate in the nation. We examined attributions of depression in clinical population of 2577 outpatients participating in mental health care in Imperial County. The aim of this study was to provide insight into how attribution of causes underlying depression may be associated with severity of depressive symptoms, and how demographic and socio-economic factors may impact attribution of depressive symptoms among rural-dwelling Hispanics, in comparison to Caucasians. Based on our review of the literature, we hypothesized that (1) Hispanics would be more likely to attribute depressive symptoms to culture-based causes (e.g., a curse or spell) than Caucasians. (2) After adjustment for acculturation, there would be no differences in how Caucasians and Hispanics attributed depressive symptoms. Additionally, we explored relationships between age, gender, education, number of years in the USA, and severity of depression within Hispanics and Caucasians. 

## 2. Methods

### 2.1. Study Sample

All subjects were outpatients assessed at Sun Valley Behavioral Medical Center—an outpatient psychiatric clinic in the town of Imperial, in California. In the 10-year period from 1995 to 2005, all patients assessed at the clinic completed an intake assessment form that included demographic information, primary complaints for which they were seeking psychiatric treatment, an acculturation questionnaire, and a questionnaire on what they attributed their symptoms to (see “Measures” below). The CESD was also administered at the time of intake. Upon assessment, DSM-III/IV diagnosis was also recorded by the treating psychiatrists. All patients were seen by one of the two psychiatrists affiliated with the facility. All records were de-identified. For this study, we identified all patients who had been given a primary diagnosis of a depressive disorder (“major depressive disorder/episode,” “depressive disorder NOS,” and “dysthymic disorder”). We excluded subjects with a diagnosis of bipolar disorder, adjustment disorder with depressed mood, or a substance-induced mood disorder. The total sample comprised 2577 subjects, of whom 1695 had a primary diagnosis of a depressive disorder. All patients were informed at the time of intake that the information provided at intake may be used for research. The study was approved by the UCSD Institutional Review Board. 

### 2.2. Measures

*Demographic Data. Demographic data* were available on participant's age, gender, years of formal education, number of years they had lived in the USA, race/ethnicity, and marital status.

*Depressive Symptom Severity.* Depressive symptom severity was assessed using the 20-item Center for Epidemiological Studies Depression Scale (CESD). The CESD is a commonly used measure of depressive symptom severity in community samples [10], with higher scores reflecting greater depression symptomatology.

*Acculturation.* The use of English language has been found to be a valid and reliable proxy measure of acculturation [4]. Hence, we measured acculturation using 5 items adapted from the acculturation rating scale for Mexican-Americans (ARSMA-II) [11]. Each item asked a specific question related to the use of English versus Spanish languages, and each item was rated from 1 (Spanish only) to 5 (English only). Higher scores indicated greater use of English. The five questions in our measure were as follows. (1) In what language do you read and speak? (2) What language did you use as a child? (3) What language do you use at home? (4) In what language do you think? (5) What language do you usually speak with friends? The mean score from these 5 items was used as the total acculturation score. The Cronbach's alpha score for the acculturation measure was 0.978, indicating excellent internal consistency.

*Attribution of Mental Items.* Based on the existing literature [12, 13], we identified four primary domains to which Mexican-Americans tend to attribute symptoms of physical and mental illness—genetic/biological factors, social/interpersonal factors, economic factors, and cultural factors. Thirteen items were selected by the primary clinician (BN) to encompass these factors in consultation with other clinical practitioners in Imperial County, and consensus was achieved regarding choice of these items. Each item was scored as either 1 (no) or 2 (yes). All questions asked whether they attributed their symptoms to a specific factor. To determine if symptoms were attributed to genetic/biological factors, we included 4 items inquiring if they attributed depressive symptoms to brain or mind problems, hereditary factors, to nutrition problems, or to drugs and alcohol. For social/personal factors, we included 3 items which assessed attribution to problems with significant others, problems with spouse, or problems with how they got along with other people. To evaluate attribution to economic factors, we included 3 items which assessed attribution to problems with their general condition, problems with finances, and job-related problems. For cultural factors, we also included 3 items that assessed attribution to curse or spell, to supernatural forces, or spiritual problems. 

### 2.3. Statistical Methods

Initially, we obtained descriptive statistics on the demographic variables and established that all persons with depressive symptoms in this sample were either Caucasian or Hispanic. We compared the demographics for the total sample to those for a subsample who had complete data, and these did not differ significantly. For subsequent analyses, we used chi-square and *t*-tests to compare the two groups on demographics, severity of depressive symptoms, acculturation scores, and the attribution items. Following this, we performed separate linear regressions within each group to determine the strongest predictors of depression severity among Hispanics and Caucasians, respectively. We selected total CESD score as the primary dependent variable and included demographics, acculturation score, and all the attribution items as independent variables. We also performed similar regression with acculturation as the primary dependent variable. All analyses were performed using SPSS version 12.0. We used Bonferroni corrections to adjust for multiple comparisons between variables, and significance level was set at 0.005.

## 3. Results

Of the total sample of 2577, 67.4% (*N* = 1104) of Hispanics and 62.8% (*N* = 561) of Caucasians were presented with some forms of depressive disorder. This difference was not significant. All comparative analyses were performed on subjects with complete data, which consisted of 1031 Hispanics and 470 Caucasians. The two groups were broadly demographically comparable. Mean age for Caucasians was 47.0 years, and for Hispanics, it was 47.5 years. Among Hispanics, 77.2% were female compared to 57.7% of Caucasians, and 49.4% of Hispanics were married compared to 39.4% of Caucasians. None of these differences attained significance. As indicated in Table 1, Caucasians had higher levels of education (average of 13 years, compared to 10 years for Hispanics), greater English acculturation, and greater number of years spent in the USA The groups did not differ on CES-D scores. On initial comparison, significantly higher numbers of Hispanics attributed depressive symptoms to “curse or spell” (*χ* ^2^ = 12.22, *P* < 0.001) or “supernatural factors” (*χ* ^2^ = 11.91, *P* < 0.001), while higher number Caucasians attributed depressive symptoms to “hereditary or genetic factors” (*χ* ^2^ = 29.80, *P* < 0.001) or “job-related stressors” (*χ* ^2^ = 10.14, *P* = 0.001).

We next performed a series of ANOVAs and chi-square tests to assess independent associations between demographic variables, acculturation and depression severity. Results of these comparisons are presented in Table 2.

We noted among both groups that rates of attribution tended to be higher among younger adults. This trend reflects relatively lower rates of depression among older adults. We also noted that among Latinos, level of education, but not years in the USA impacted attribution. In both groups, the association with marital status was low. Biological attributions such as alcohol or drugs, problems with brain or mind, diet (vitamins/nutrients), or hereditary factors seemed to be significant only in persons with relatively higher severity. Culture-based variables such as attribution of depression to curses/spells, supernatural factors, or spiritual factors were more prominent among Latinos with lower levels of education. On the other hand, attribution to factors such as job stress or financial issues was more prominent among more educated persons in both groups. Among Caucasians, associations with age and years in the USA paralleled each other, since most persons had lived in the USA all their lives. To gain a clearer understanding of how acculturation may impact attribution, we conducted linear regression with acculturation as the primary dependent variable (Table 3) and noted that it was not associated with any attribution item among Caucasians after adjusting for age, gender, years in the USA or marital status. Among Latinos, lower acculturation predicted likelihood of attribution of symptoms to “curse or spell,” and higher acculturation predicted higher likelihood of attribution to “job” or “hereditary factors.”

Next, we conducted separate within-groups linear regressions to assess independent associations between depression severity and attribution after adjusting for demographic variables (Table 4). After adjusting for age, gender years of schooling, years in the USA, marital status, and level of acculturation among both Hispanics and Caucasians, higher CESD scores were predicted by attribution of symptoms to “problems with significant others” and “problems with how they got along with people.” 

Among Hispanics, however, there was additional significant predictive association between CESD scores and “problems with job situation” and “problems with finances.” The association with curse/spell and other supernatural factors did not remain significant after adjustment for demographic and acculturation items. 

## 4. Discussion

This study examined how rural Hispanics and Caucasians attributed depressive symptoms that led them to seek psychiatric care. We noted that there was no difference between groups in severity of depression at time of outpatient intake. However, Hispanics were almost twice as likely as Caucasians to attribute their depression to a curse or spell or supernatural factors, thus confirming our first hypothesis, even though fewer than 10% of both groups actually attributed depressive symptoms to these culture-related factors. We also noted that Caucasians were more likely to attribute depressive symptoms to hereditary or genetic factors or to job-related reasons. This finding may reflect the higher education levels among the Caucasian patients. It may also reflect a tendency for persons with higher education and socioeconomic status to attribute their problems to causes related to a biological or social factors, rather than psychological causes [14]. In our subsequent within-group comparison of the two groups, we noted that that after adjusting for income, age, and education, severity of depression was predicted by attribution to problems with significant others or other people in both groups. Additionally, among Hispanics, severity of depressive symptoms was predicted by problems with their job situation or finances. Thus, our second hypothesis was not confirmed. This indicates that overall, there was a greater tendency to attribute depressive symptoms to extrinsic (social) factors, rather than intrinsic (biological) factors. It also indicates that among Hispanics, finances and employment may be more prevalent in causing the initiation of treatment than among Caucasians. Since the Hispanic population is Imperial County, is less affluent, copes with record-high unemployment, and has greater needs, this finding echoes similar findings in other populations that deal with a great burden of psychosocial stress [15]. Our finding hints at depression being one possible outcome of chronic psychosocial stress. Jimenez et al. [16] have previously described how Latinos tended to attribute depression to psychosocial stressors such as problems with family and relocation. While we did not measure relocation, our findings echo their suggestion that stress resulting from the environment or life situation may be the primary attribute of depression. We also noted that over 60% of Hispanics attributed depression to “brain/mind problems.” This high rate likely reflects a sampling bias, since these were psychiatry outpatients. However, these associations must be interpreted with caution, considering that there is evidence suggesting optimism from being immigrants and culture-related resilience may also have protective roles, especially among rural populations. Our study was not designed to study impact of these factors [17]. 

It is also important to note other limitations of this study. The data were based on retrospective chart review, did not employ stringent diagnostic criteria, and did not incorporate information gathered from subsequent clinical visits or prior treatment records (i.e. they are cross-sectional). In addition, we did not have data to test the impact of potentially relevant clinical and demographic factors such as ethnic origins, belonging to specific subgroups, impact of medications, and previous treatments. While we believe that utilizing an existing theoretical or explanatory model may have enabled us to draw more precise conclusions, available data did not allow us to adequately test or modify the existing models addressing this topic. Further, since all questionnaires were self-reported, they may be impacted by recall and reporting biases, which are influenced by culture and stigma to mental illness. Since all patients were seen in the same outpatient clinic and had some form of health insurance, our population may not represent the overall USA population of rural-dwelling Hispanics. However, since all the patients were examined by one of the two psychiatrists, we believe that there is consistency of diagnostic methods across the sample. We were unable to study the impact of income, which may have confounded some findings. Moreover, since our sample comprised entirely of persons who were legal residents of the USA and had access to healthcare, this population may not be representative of the entire population of the Imperial County. Finally, while the differences we observed represent statistical significance, the overall clinical significance is difficult to establish. This may vary from individual to individual. 

Nevertheless, the interaction of depressive symptoms, attribution, acculturation, and demographics has implications for healthcare delivery as the USA population adds more Hispanics, especially in areas along the U.S.-Mexico Border Region. We intend that this study sheds some preliminary light on the interplay of stressors and depressive symptoms in the population along the border region, and we recommend that future studies employ longitudinal design and broader use of validated measures and be more inclusive of culture-related and psychosocial factors that are likely to impact attribution and severity of depressive symptoms in this population.

## Figures and Tables

**Table 1:** Comparison between demographic traits and rates of attribution between Hispanics and Caucasians.

Variable	Hispanics	Caucasians	*F*/*χ* ^2^	*P* value
	Mean (Std Dev)/*N* (%)	Mean (Std Dev)/*N* (%)		
*N*	1031	470	—	—
Age (years)	47.0 (17.1)	47.5 (14.7)	0.33	0.564
Gender (female)	796 (77.2%)	271 (57.7%)	1.34	0.246
Married	464 (45.4%)	185 (39.4%)	4.8	0.029
Education (years)	10.3 (5.0)	13.5 (3.5)	159.4	<0.001*
Years in the USA	31.5 (17.3)	46.6 (15.7)	260.4	<0.001*
Acculturation scale	12.5 (6.8)	23.9 (2.6)	1132.1	<0.001*
CESD total	19.9 (19.1)	18.9 (18.8)	0.8	0.371

Attribution items (affirmative attribution)				

Significant others	471 (45.7%)	200 (42.5%)	1.34	0.246
Other family members	501 (48.6%)	198 (42.2%)	5.06	0.024
Curse/spell	108 (10.5%)	24 (5.0%)	12.22	<0.001*
General condition	693 (67.2%)	346 (73.7%)	6.24	0.012
Hereditary factors	371 (36.0%)	240 (51.1%)	29.80	<0.001*
Job related	392 (32.8%)	195 (41.4%)	10.14	0.001*
Supernatural	93 (9.0%)	18 (3.9%)	11.91	0.001*
Getting along with others	342 (33.2%)	172 (36.6%)	1.62	0.203
Brain/mind	626 (60.7%)	293 (62.4%)	0.37	0.540
Finances	493 (47.8%)	211 (44.8%)	1.12	0.290
Spiritual problems	245 (23.8%)	99 (21.1%)	1.30	0.254
Lack of nutrients	419 (40.6%)	162 (34.5%)	4.89	0.020
Alcohol/drugs	97 (9.4%)	40 (8.6%)	0.23	0.027

*Significant after applying Bonferroni correction.

**Table 2:** Associations between sociodemographic variables, clinical variables, and attribution among Latinos.

	Older		Male		Greater		More years		Being		Greater		Higher CESD	
	age		gender		education		in USA		married		acculturation		score	
	Lat	Cau	Lat	Cau	Lat	Cau	Lat	Cau	Lat	Cau	Lat	Cau	Lat	Cau
Significant others	−	−			+		−	−	+		+	−	+	
Other family members	−	−	−	−				−					+	+
Curse/spell			+		−									
General condition	−	−			+			−			+		+	+
Hereditary factors	−	−			+	+		−			+		+	+
Job related	−	−	+		+	+		−			+			+
Supernatural			+	+	−									
Getting along with others	−	−	+		+			−			+		+	+
Brain/mind													+	+
Finances	−	−			+		−	−	−	−			+	+
Spiritual problems	−	−						−					+	+
Lack of nutrients		−						−					+	+
Alcohol/drugs	−		+	+	+						+			

Age, education, years in the USA, acculturation, and CESD scores were compared to attribution using ANOVA. Gender and marital status were compared using chi-square tests.

Lat: Latino; Cau: Caucasian.

+ indicates a statistically significant positive association (*P* < 0.005).

− indicates a statistically significant negative association (*P* < 0.005).

Empty cells indicate statistically insignificant correlation of means.

**Table 3:** Within-group linear regressions predicting acculturation.

Attribution of symptoms	Hispanics		Caucasians	
	*B*	*P* value	*B*	*P* value
Significant others	0.35	0.26	−0.05	0.83
Other family members	0.14	0.65	−0.24	0.27
Curse or spell	−1.57	0.001	−0.14	0.74
General condition	0.22	0.50	0.19	0.42
Hereditary factors	1.06	0.001	0.24	0.25
Job related	1.12	0.001	0.03	0.90
Supernatural	0.27	0.61	−0.82	0.08
How you get along with people	0.18	0.60	−0.03	0.89
Brain/mind	0.10	0.76	0.09	0.70
Finances	−0.84	0.006	−0.14	0.51
Spiritual factors	0.002	0.99	0.08	0.77
Lack of nutrients	−0.03	0.93	−0.25	0.25
Alcohol/drugs	1.25	0.005	0.11	0.70

All comparisons were adjusted for age, gender, years of schooling, years in the USA, and marital status.

**Table 4:** Within-group linear regressions predicting CESD scores.

Attribution of symptoms	Hispanics		Caucasians	
	*B*	*P* value	*B*	*P* value
Significant others	2.04	0.001	2.33	0.006
Other family members	0.16	0.804	1.42	0.085
Curse or spell	1.76	0.170	0.58	0.780
General condition	0.78	0.254	1.46	0.091
Hereditary factors	0.45	0.527	1.61	0.066
Job related	1.49	0.026	1.46	0.063
Supernatural	−2.30	0.081	−1.54	0.463
How you get along with people	1.85	0.012	2.07	0.033
Brain/mind	1.07	0.105	1.23	0.143
Finances	1.74	0.007	1.26	0.141
Spiritual factors	0.42	0.618	1.74	0.134
Lack of nutrients	0.40	0.630	1.22	0.243
Alcohol/drugs	1.43	0.180	0.50	0.698

All comparisons were adjusted for age, gender, years of schooling, years in the USA, marital status, and level of acculturation.
